# The effect of amorphous silica on soil–plant–water relations in soils with contrasting textures

**DOI:** 10.1038/s41598-024-60947-1

**Published:** 2024-05-04

**Authors:** Mohsen Zarebanadkouki, Wael Al Hamwi, Mohanned Abdalla, Rasoul Rahnemaie, Jörg Schaller

**Affiliations:** 1 Professorship for Soil Biophysics and Environmental Systems, Technical University of Munich, Munich, Germany; 2 Leibniz Center for Agricultural Landscape Research (ZALF), Müncheberg, Germany; 3 Chair of Root–Soil Interaction, School of Life Sciences, Technical University of Munich, Munich, Germany; 4 Department of Soil Science, Tarbiat Modares University, Tehran, Iran

**Keywords:** Amorphous silica, Drought, Leaf water potential, Soil amendment, Soil hydraulic conductivity, Soil–plant hydraulic model, Agroecology, Hydrology

## Abstract

This study investigates how amorphous silica (ASi) influences soil–plant–water interactions in distinct soil textures. A sandy loam and silty clay soil were mixed with 0 and 2% ASi, and their impact on soil retention and soil hydraulic conductivity curves were determined. In parallel, tomato plants (*Solanum lycopersicum* L.) were grown in experimental pots under controlled conditions. When plants were established, the soil was saturated, and a controlled drying cycle ensued until plants reached their wilting points. Soil water content, soil water potential, plant transpiration rate, and leaf water potential were monitored during this process. Results indicate a positive impact of ASi on the sandy loam soil, enhancing soil water content at field capacity (FC, factor of 1.3 times) and at permanent wilting point (PWP, a factor of 3.5 times), while its effect in silty clay loam was negligible (< 1.05 times). In addition, the presence of ASi prevented a significant drop in soil hydraulic conductivity (${K}_{h}$) at dry conditions. The ${K}_{h}$ of ASi-treated sandy loam and silty clay at PWP were 4.3 times higher than their respective control. Transpiration rates in plants grown in ASi-treated sandy loam soil under soil drying conditions were higher than in the control, attributed to improved soil hydraulic conductivity. At the same time, no significant difference was observed in the transpiration of plants treated with ASi in silty clay soil. This suggests ASi boosts soil–plant–water relationships in coarse-textured soils by maintaining heightened hydraulic conductivity, with no significant effect on fine-textured soils.

## Introduction

The hydraulic conductivity of both the soil and roots significantly influences water movement from the soil to the roots. In wet soil conditions, the hydraulic conductivity of the roots is the primary factor in determining the water flow into the roots. However, as the soil dries, its hydraulic conductivity decreases dramatically, potentially limiting water uptake by the roots^1,2^. This reduction varies depending on soil texture, with coarse-textured soils showing a more significant decrease than fine-textured soils Modeling studies have demonstrated that as the soil dries, water potential drops significantly in the vicinity of roots (referred to as the rhizosphere), resulting from the radial flow of water towards the roots and the non-linear nature of soil conductivity in unsaturated conditions^3–5^. This drop in soil water potential requires a concomitant drop in leaf water potential to maintain transpiration rates. Because hydraulic conductance declines with increasingly negative water potential, plants will close stomata and curb the drop in leaf water potential to the extent that depends on species, e.g., isohydric vs. anisohydric behavior^6^. Several studies have provided experimental proof that transpiration reduction is triggered by the loss of hydraulic conductivities across the soil–plant system^7–9^, especially the soil–root system^10,11^.

The ability of soil to retain water is primarily determined by its pore size distribution and specific surface area. Generally, soils with a coarser texture exhibit lower water-holding capacity than those with a finer texture. Several practices have been proposed to improve soil hydraulic properties, including using organic amendments, such as livestock manure, compost, plant residues, and biochar^12–15^. Studies have shown that these amendments can enhance water retention and flow within soils by impacting soil aggregation, pore size distribution, and water adsorption properties, owing to their high specific surface area and water-holding capacity. Mineral-based amendments, like natural zeolites, are also being investigated for their high internal porosity and low bulk density^16^. Similarly, synthetic components with high water-holding capacity, such as superabsorbent polymers, have been evaluated and found to improve water retention and flow within soils^17^. This promising technique holds significant potential for increasing the water-holding capacity of soils, especially in regions that face water scarcity.

In recent years, several studies have suggested that the addition of amorphous silica (ASi) to soils may improve soil–plant–water relationships by enhancing the water-holding capacity of soils^18,19^, which has been attributed to the high surface area of ASi, along with its high adsorption potential, and its high internal porosity. Furthermore, the addition of ASi to the soils has been shown to positively affect the hydraulic conductivity of coarse-textured soils, enabling them to maintain high conductivity levels even as the soil dries^18,19^. Field experiments on sandy soils treated with ASi have also reported increased soil moisture levels throughout the wheat growing season^20^. These observations suggest that adding ASi to soils could improve soil–plant–water relations during soil drying cycles by increasing water availability for plant uptake and maintaining a higher water flux to the roots under drying conditions by influencing the hydraulic conductivity of soils. However, to date, there is limited experimental evidence exploring the effect of ASi on soil–plant–water relations, such as plant transpiration rate, stomatal conductance, and leaf xylem water potential during soil drying cycles, in particular in soils with varying textures^20,21^. Moreover, there is limited evidence of the impact of ASi on the hydraulic properties of fine-textured soils and soil–plant–water relations in these soils. Although several studies have reported enhanced plant performance after Si fertilization (primarily as silicic acid, which precipitates as ASi as the soil dries) and thus will exhibit the same effects on soil water relations as ASi^22^ under drought conditions by improving the photosynthetic rate and, in most cases, stomatal conductance of drought-stressed plants, but those studies have not measured the effect of Si on the hydraulic properties of soils^23–26^. Therefore, further research is required to investigate the potential benefits of ASi on soil–plant–water relations, particularly during soil drying cycles, and its impact on the hydraulic properties of coarse and fine-textured soils. Such studies will be instrumental in providing insights into the mechanisms by which ASi influences soil–plant–water relationships and identifying its potential as a sustainable solution to improve soil water-holding capacity and mitigate the impact of water scarcity on agricultural production.

This study examines the impact of adding ASi into soils with varying textures on the soil–plant–water relations atmosphere continuum. We hypothesize that ASi, by enhancing the retention and flow of water within soils, may facilitate plant access to water during soil drying, the magnitude of which is expected to be soil texture dependent. To test this hypothesis, we grew tomato plants (*Solanum lycopersicum* L.), known to exclude silicon uptake and have low silicon accumulation in aboveground biomass^27^, in two contrasting soil textures (coarse and fine soils). The tomato plant was selected to exclude the potential impact of ASi on water relations within plants, allowing us to evaluate its effect exclusively on the hydraulic properties of soils. Both soil types were treated with and without ASi, and the plants were grown in a climate chamber and watered regularly. Once the plants were well established, the soil was saturated with water and then allowed to dry, during which soil and plant-related water properties were quantified. In parallel, the effect of ASi on the hydraulic properties of both soils was determined.

## Results

### Effect of Si on hydraulic properties of the two selected soils

**Figure 1:**
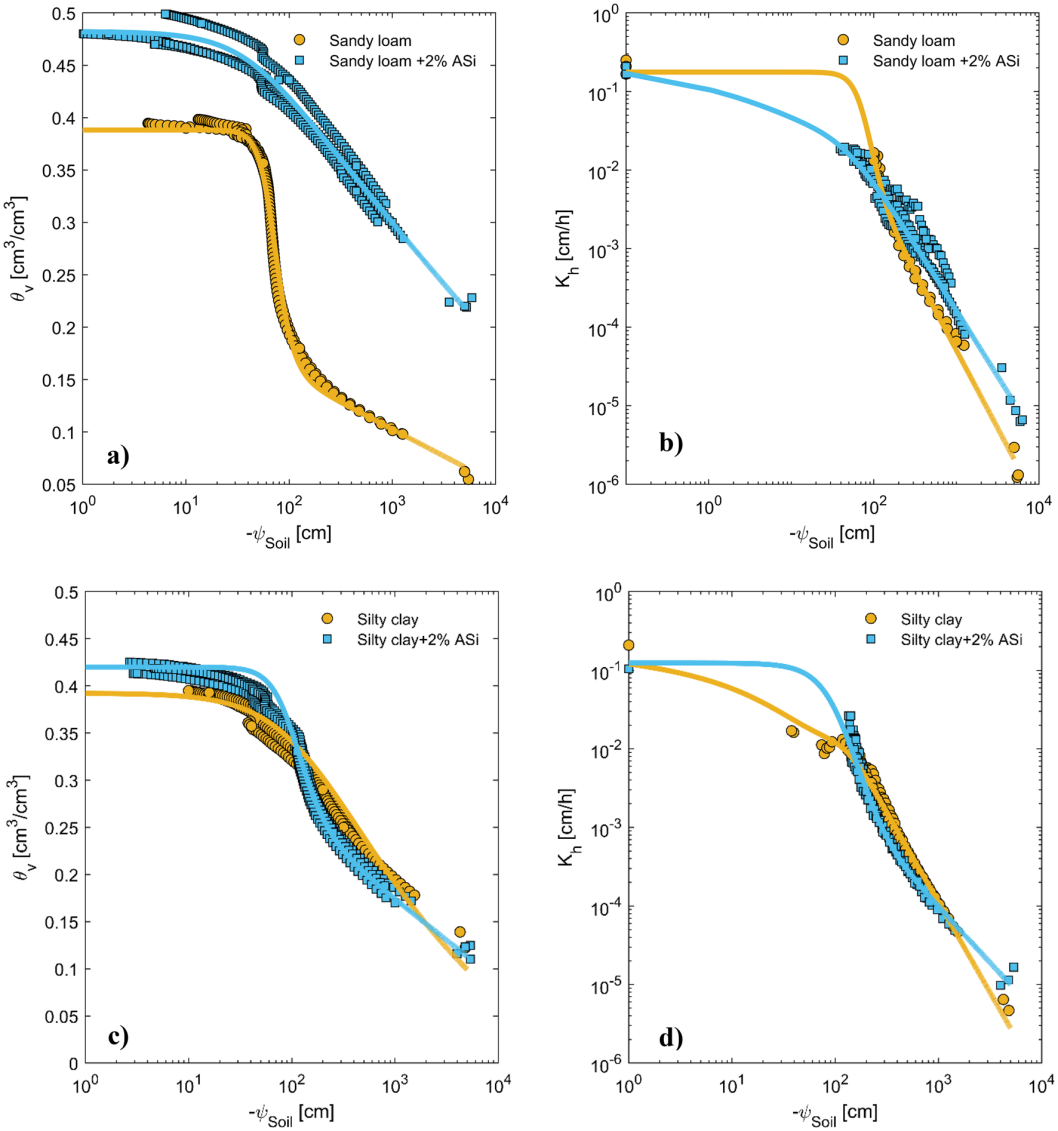
Soil retention curves (**a,c**) and hydraulic conductivity curves (**b,d**) of sandy loam and silty clay soil mixed with different Si contents (0: control and 2% ASi). The dots show the measurements of three replications, and the solid lines show the best-fitted curve according to Peters–Durner–Iden model^34^ among three replications together. The effects of Asi on soil water content and hydraulic conductivity at selected soil matric potentials are statistically analyzed in Table 1.

Figure 1 provides a comprehensive analysis of the impact of ASi on soil water retention and hydraulic conductivity curves of two selected soils in this study. The data were analyzed using the PDI model, and the best-fitted line was plotted to compare the results between the two soils. The data presented in Table 1 further extends the analysis by providing information on the water content and hydraulic conductivity of the soils at various soil matric potentials. Water content at the field capacity (FC) and permanent wilting point (PWP) were respectively 0.33 and 0.05 cm^3^ cm^−3^ in the control treatment of sandy loam and increased statistically significant to 0.44 and 0.18 cm^3^ cm^−3^ in sandy loam soil treated with 2% ASi (p = 0.01 and average of three replication). These increases were a factor of 1.33 and 3.5 times for FC and PWP, respectively. Although water contents in the FC and PWP were increased in the sandy loam, the presence of ASi had no significant effect on the plant-available water content AWC, defined as the difference between FC and PWP (0.28 vs 0.27 cm^3^ cm^−3^). The control silty clay soil had FC and PWP of 0.36 and 0.10 cm^3^ cm^−3^, which were 0.38 and 0.1 cm^3^ cm^−3^, respectively, in the presence of 2% ASi. In other words, ASi increased the FC of silty clay soil by 1.05 times, which was statistically significant at p = 0.05 and had no effect on PWP. In contrast to the case of sandy loam, the overall impact of ASi on AWC was statistically significant (an increase by a factor of 1.07 times). The analysis of the soil hydraulic conductivity curve further revealed that adding ASi significantly impacts water movement within the soil. Specifically, adding ASi to sandy loam soil reduced the drop in hydraulic conductivity as the soil dried, resulting in a higher hydraulic conductivity than the control. The addition of ASi to sandy loam soil reduced its hydraulic conductivity by a factor of 10 at FC but maintained it 4 times higher than the control as soil dried (Table 1). Adding 2% ASi to the silty clay soil maintained its hydraulic conductivity 20 and 7 times higher than the control at FC and PWP, respectively (Table 1). Overall, the results indicated that the addition of ASi significantly impacts soil water content and hydraulic conductivity, varying depending on the soil texture and matric potential (Table 1).

**Table 1:** Effect of ASi on water content and hydraulic conductivity of different soils at varying soil matric potential ranges, namely field capacity (FC), matric potential of 4000 cm (h4000), permanent wilting point (PWP), and plant available water content (AWC).

Parameter	Sandy loam		Silty clay	
	Control	+ 2% ASi	Control	+ 2% ASi
Soil water contents [cm^3^ cm^−3^]				
FC	0.33 ± 0.005	0.44 ± 0.01**	0.36 ± 0.01	0.38 ± 0.01*
h4000	0.07 ± 0.01	0.28 ± 0.05**	0.25 ± 0.00	0.23 ± 0.01*
PWP	0.05 ± 0.01	0.18 ± 0.01**	0.1 ± 0.01	0.1 ± 0.01ns
AWC	0.28 ± 0.01	0.27 ± 0.00ns	0.26 ± 0.00	0.28 ± 0.00*
Soil hydraulic conductivity [cm h^−1^]				
FC	0.24 ± 0.14	10.01 ± 0.01ns	2.9E−02 ± 6.7E−03	0.68 ± 0.9ns
h4000	4.E−06 ± 1.4E−06	2.1E−05 ± 4.5E−06*	9.9E−06 ± 1.1E−06	1.8E−05 ± 3.6E−06*
PWP	2.2E−07 ± 5.1E−08	9.4E−07 ± 3.3E−07*	3.2E−07 ± 5.8E−08	2.4E−06 ± 1.1E−06*

The data represents the average of three replications and includes the standard deviation to provide a comprehensive understanding of the variability in the results.

The symbols *, ** and ns refers to significant different in level p = 0.05, p = 0.01 and no significance difference, respectively evaluated b t-test.

### Effect of ASi on soil–plant–water relation

**Figure 2:**
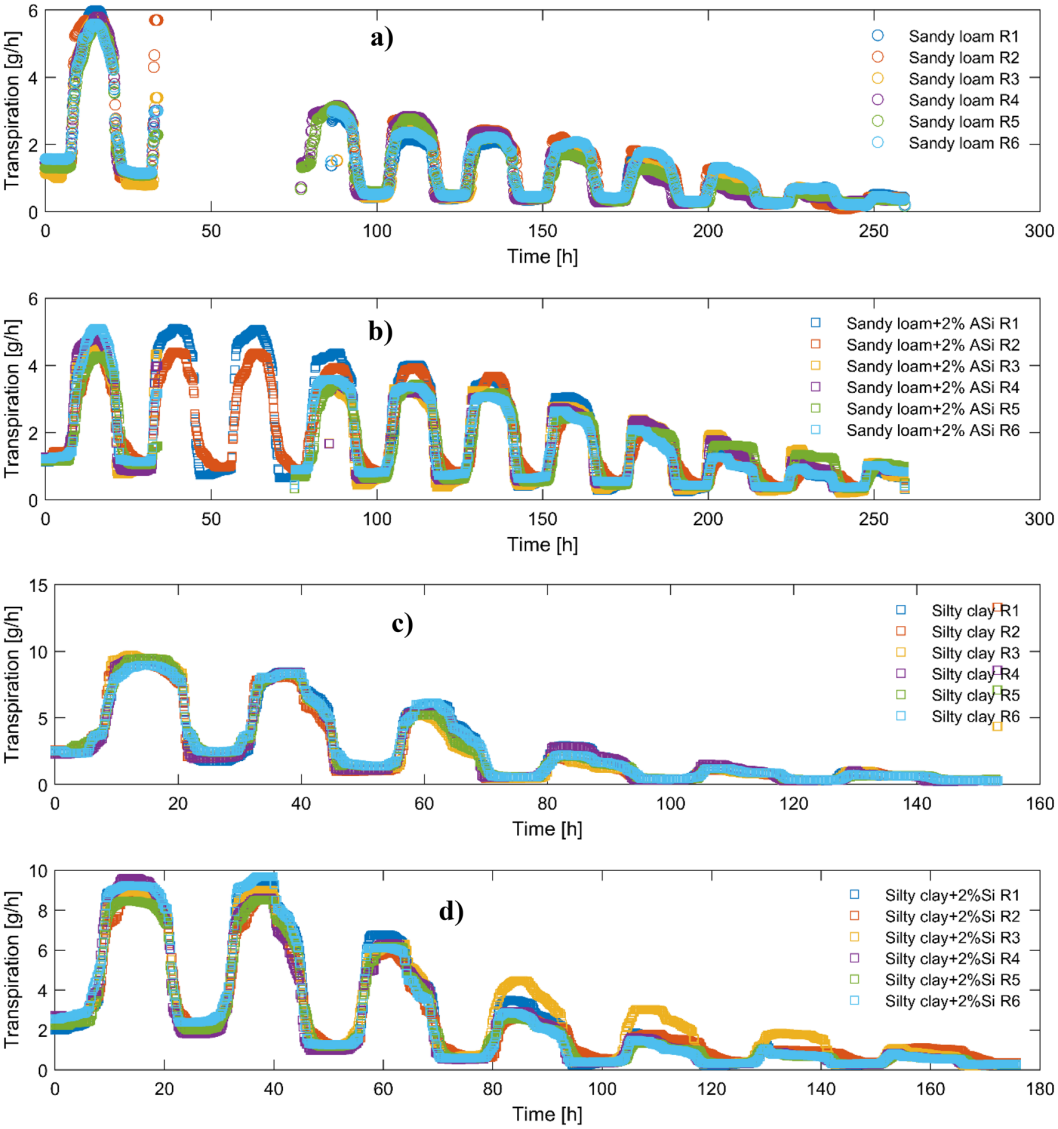
Plant transpiration over time during a soil drying cycle in two different soils: control sandy loam and silty clay soil (**a,c**) and sandy loam and silty clay soil treated with 2% of ASi (**b,d**). The different colors represent different replications. Time zero refers to 12 h after the last irrigation. The absence of transpiration data on the second day of the drying cycle is due to the malfunctioning of respective balances in recording the weights.

The transpiration rates of plants grown in sandy loam soil with varying ASi content are presented with time during a soil drying cycle in Fig. 2. As expected, the initial transpiration rates were high immediately after irrigation, followed by a decline as the soil dried over time. However, when comparing the transpiration rates at the end of the soil drying cycle, the data suggests that the plants grown in the soil treated with ASi sustained a higher transpiration rate than the control soil. This observation may indicate an improvement in the plant’s access to water in the treated soil, but it could also be due to the more significant amount of water stored in the treated soil after irrigation. Note that the later comparison cannot be easily analyzed for its significant difference since we cannot compare different replications at the same water contents.

**Figure 3:**
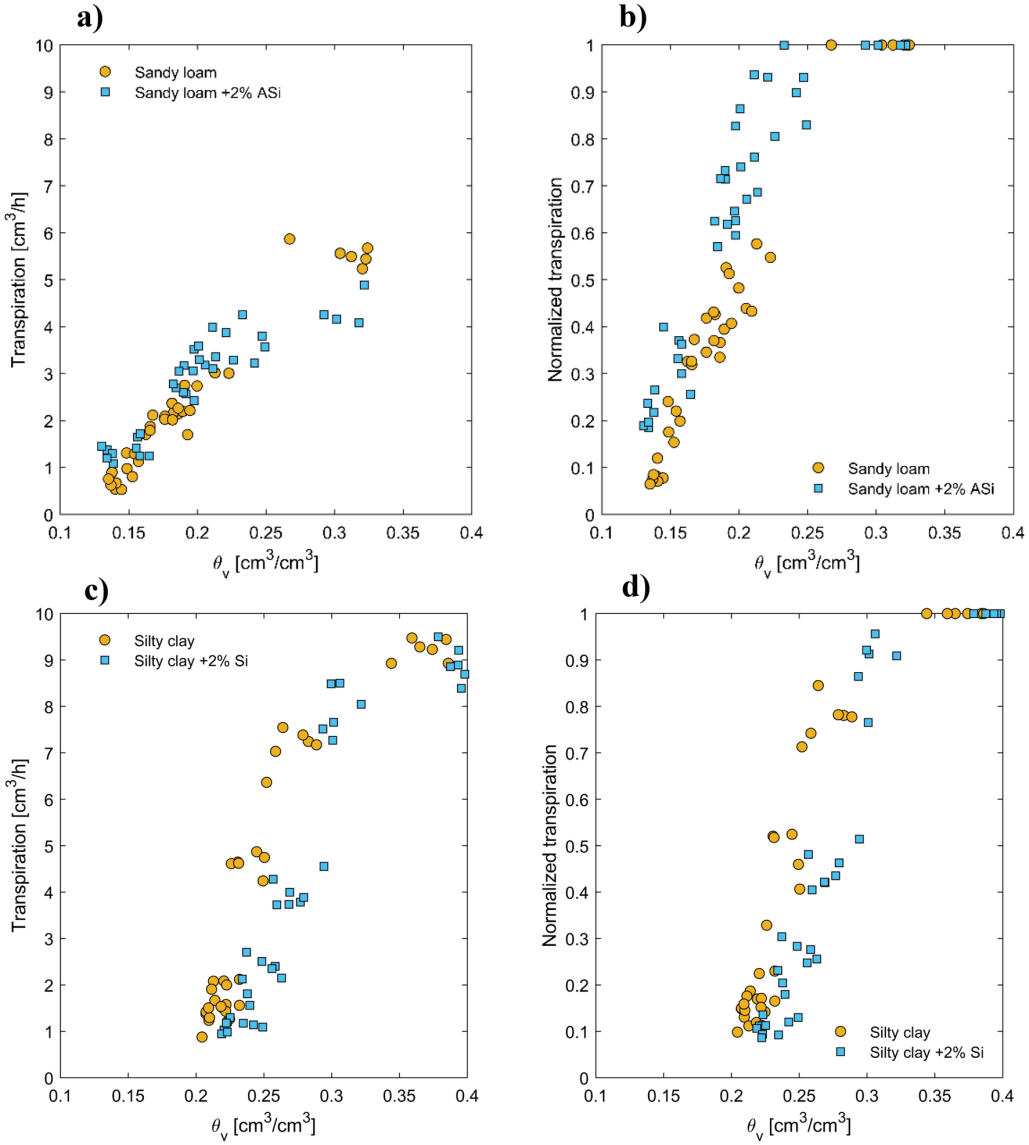
Left: midday plant transpiration as a function of soil water content for four different tested soils. The dots represent data from six replications and include measurements taken at the peak of plant transpiration, which occurs around midday. The graph on the right shows normalized midday plant transpiration as a function of soil water content. In this graph, midday plant transpiration values are normalized by the maximum plant transpiration measured on the first day of the soil drying cycle.

The data for midday plant transpiration as a function of soil water content is presented in Fig. 3. After the last irrigation, the control soil and soil mixed with 2% of ASi had similar water content at the beginning of the soil drying cycle. Although midday plant transpiration in both soils was reduced as the soil dried, those mixed with ASi showed slightly lower transpiration rates than the control soil in the wet range, but the opposite trend was observed in the dry range.

**Figure 4:**
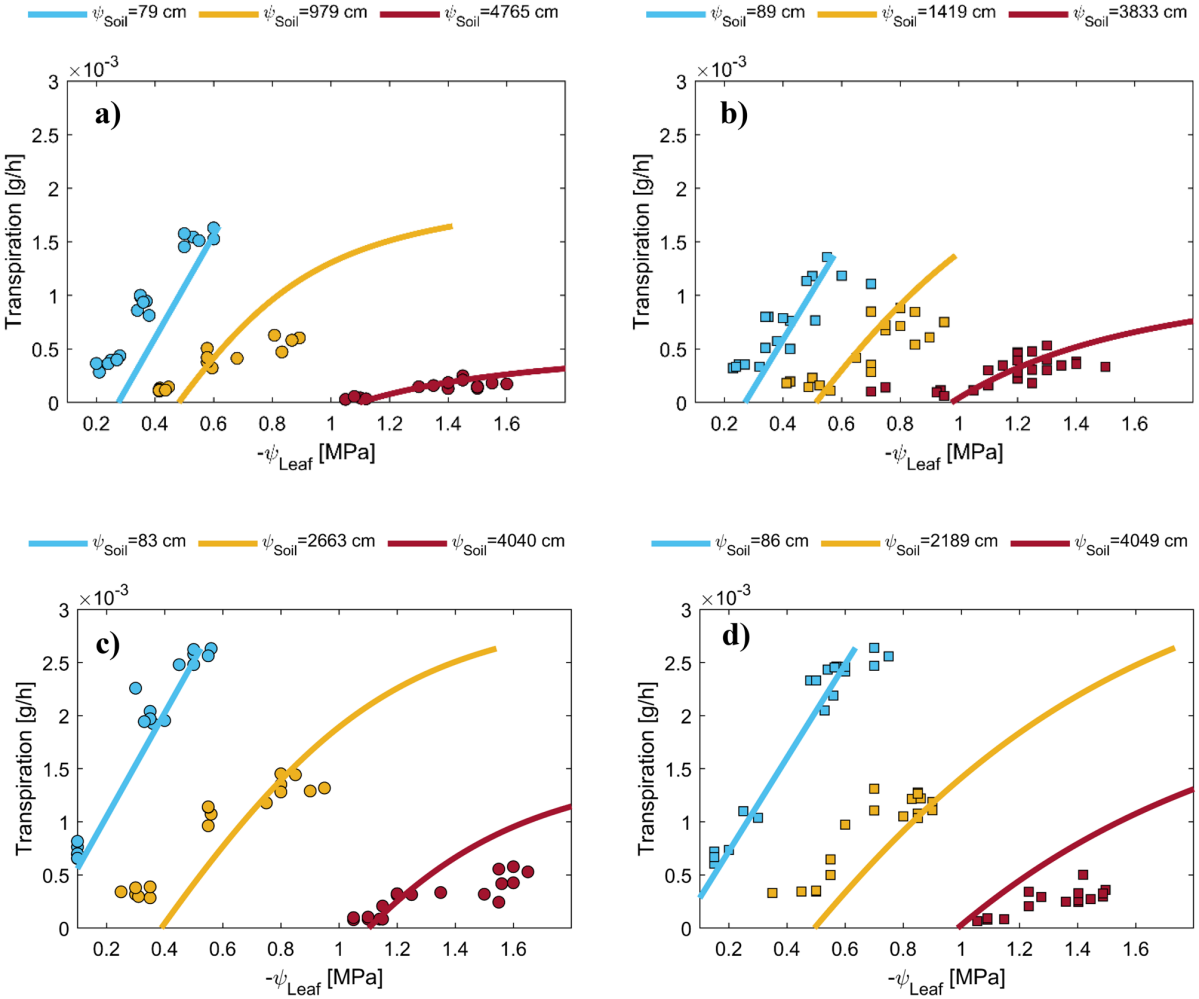
The relationships between plant transpiration and plant xylem water potential were measured (represented by dots) and simulated (represented by lines) at three selected soil water potentials for four different soil types: sandy loamy (**a**), sandy loam mixed with 2% ASi (**b**), silty clay (**c**), and silty clay mixed with 2% ASi (**d**). The dots represent data from six replications and include measurements taken at night, in the morning, and at midday for a given soil water content. The legends indicate the average soil matric potential among the six replications measured at nighttime.

**Figure 5:**
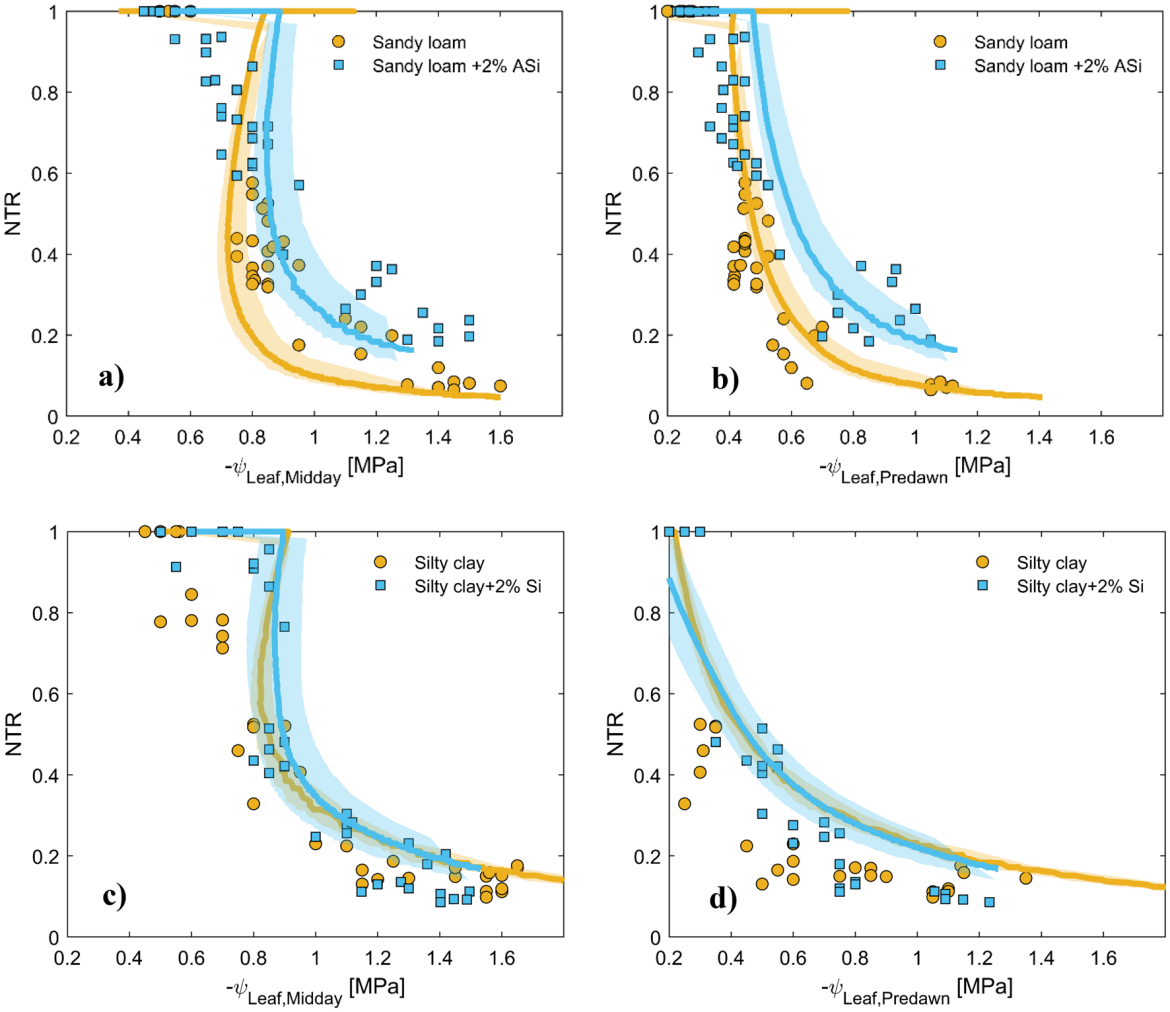
(**a,c**) impact of ASi content on normalized midday plant transpiration (NTR) and midday plant xylem water potential (${\psi }_{Leaf.Midday}$) in sandy loamy and silty clay soils. (**b,d**) impact of ASi content on normalized midday plant transpiration (NTR) and predawn xylem water potential (${\psi }_{Leaf, Predawn}).$ The data pertains to a soil drying cycle, and dots represent measurements of six different plants. The gray band surrounding the fitted lines indicates the 95% confidence interval of the fittings. If the confidence intervals do not overlap, we assumed that there is a significant difference between two tested variables. The predawn leaf xylem water potential is taken as proxy of soil water potential. This is because the water potential within the plant xylem is assumed to approach the soil water potential at relatively lower observed transpiration rates during the night. The effect of Asi on ${\psi }_{Leaf.Midday}$ and ${\psi }_{Leaf, Predawn}$ at some selected NTRs are statistically analyzed in Fig. 6.

**Figure 6:**
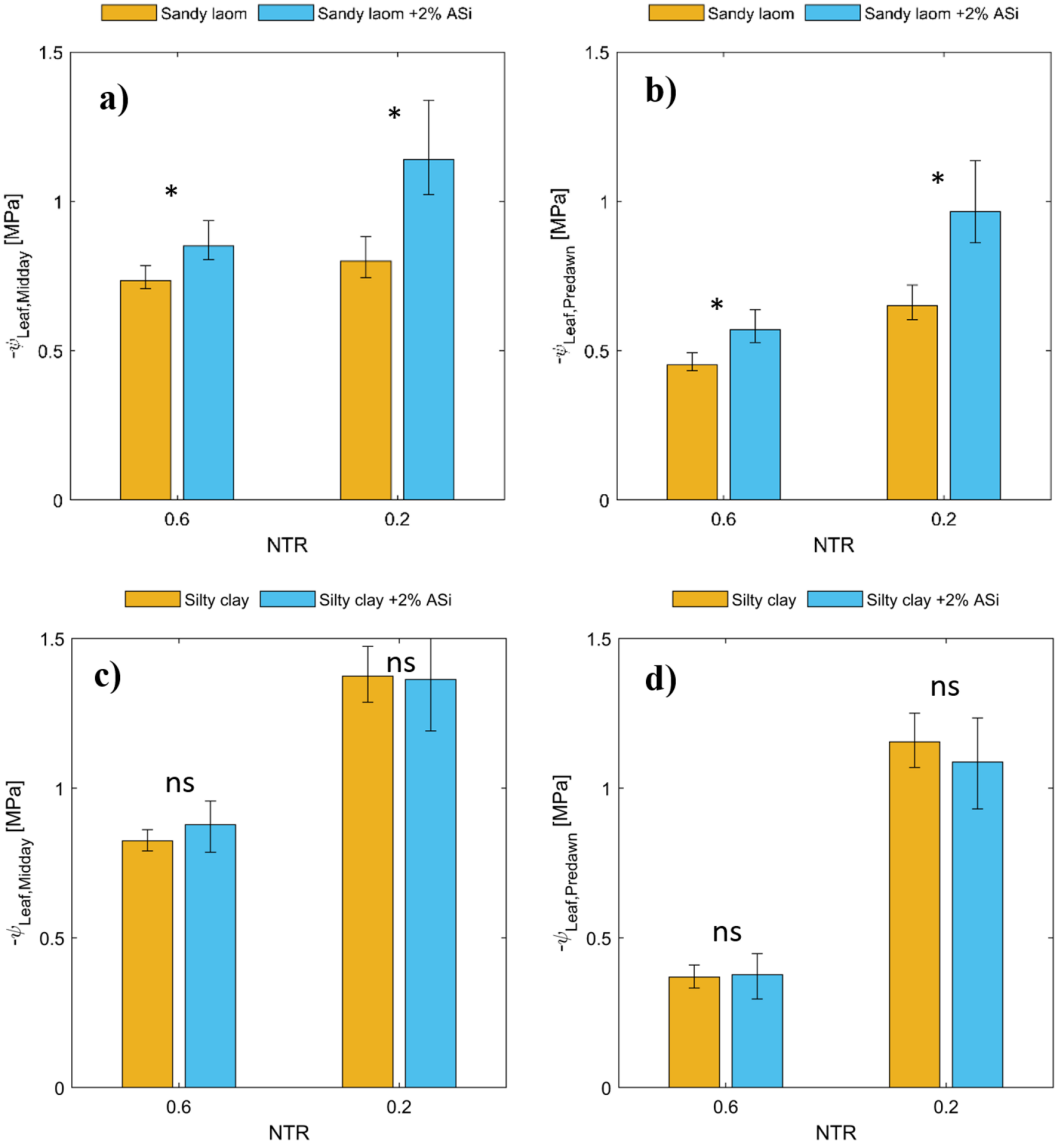
The effect of ASi content at two selected normalized transpiration (NTR) of 0.6 and 0.2 on predawn xylem water potential $({\psi }_{Leaf, Predawn}$) and midday plant xylem water potential $({\psi }_{Leaf.Midday})$ in sandy loamy (**a,b**) and silty clay soil (**c,d**). The data are obtained from the best line through measured relations. The error bars show the 95% confidence interval of the fittings. If the confidence intervals do not overlap, we assumed that there is a significant difference p = 0.5 between at two tested variables as shown with * and other was difference is not significant as shown with ns. The predawn leaf xylem water potential is taken as a proxy of soil water potential.

We simulated soil–plant–water relations using a simple soil–plant hydraulic model. This allowed us to reproduce a continuous relationship between soil water potential/content, leaf water potential, and plant transpiration rate, which was needed to compare plant transpiration rate and leaf water potentials at similar soil water contents/potentials. Note that our measurements would not necessarily provide this option. Figure 4 displays the measured and simulated relationships between the plant transpiration rate and the water potential in plant xylem under three different soil matric potentials. To match the relationships between the soil water potential, leaf water potential, and the varying plant transpiration rate, we performed an inverse modeling exercise by adjusting the active length of roots involved in water uptake. This model has been used in several papers, and its assumptions have been discussed elsewhere^10,28^. Our results indicated that our model could reproduce the observed relationship between the plant transpiration rate and the xylem water potential at the three selected soil water potentials (Fig. 5). Specifically, we noted a linear relationship between the plant transpiration rate and the xylem water potential at high soil matric potentials (wet soil range). In contrast, the relationship became nonlinear as the soil dried (low soil matric potential). To better illustrate the distinct differences observed in the tested treatments, we first identified the onset of a nonlinear relationship between simulated plant transpiration and xylem water potential. The procedure of identifying the onset of nonlinearity was detained in materials and methods. The plant transpiration rate at the onset of nonlinearity was assumed to correspond to the maximum observed transpiration by plants at any given soil matric potential, serving as a critical indicator for the following analysis. Figure 5a,c present the relationship between simulated and measured plant-normalized midday transpiration rates (NTR) and plant midday leaf xylem water potential throughout the soil drying cycle. The NTR was obtained by dividing the transpiration rate at any measured time by the maximum observed transpiration of the respective plant on the first day of the drying cycle when the soil was still wet. The results revealed a fascinating contrast in the behavior of sandy soil with the presence of ASi. The results showed that in the case of sandy soil presence of ASi in the soils delayed the onset of nonlinearly in the relation between plant normalized transpiration and xylem water potential (occurring in more negative leaf water potential) and also allowed plants to sustain higher transpiration while experiencing a given leaf water potential under conditions where transpiration was constrained by soil drying (Fig. 5a). However, the ASi did not significantly affect the relationship between normalized transpiration rate and xylem water potential in silty clay soil (Fig. 5c). This observation implies that ASi's influence on plant water relations might be soil texture dependent. To further understand the effects of ASi, two normalized transpiration (NTR) of 0.6 and 0.2 were selected and data of predawn xylem water potential $({\psi }_{Leaf, Predawn}$) and midday plant xylem water potential $\left({\psi }_{Leaf,Midday}\right)$ are presented in Fig. 6. At these two selected NTRs both ${\psi }_{Leaf, Predawn}$ and ${\psi }_{Leaf,Midday}$ were significantly higher in sandy loam treated with ASi than control.

## Discussion

This study aimed to investigate the effects of ASi application on the retention and flow of water in soils with different textures and how, in turn, ASi application may affect plant access to water under soil drying conditions. Our findings showed that the application of ASi significantly impacted the retention and flow of water in sandy loam soil (representing coarse-textured soil). In contrast, its effect in silty clay soil was less pronounced (representing fine-textured soil. Moreover, the results revealed that using ASi improved soil–plant water relations in sandy loam soil, resulting in a higher transpiration rate in tomato plants under soil drying conditions compared to the control treatment. Specifically, tomato plants grown in sandy loam soil treated with 2% ASi could sustain a higher transpiration rate than those grown in the control treatment.

The findings of this study underscore the substantial impact of applying ASi on the water content at FC and PWP in sandy loam soil (Fig. 1, Table 1), similar to the trend often observed by the application of soil amendments such as compost, natural zeolites, and superabsorbent polymers^16,17^. The presence of ASi resulted in a 1.33-fold increase in water content at FC and a remarkable 3.5-fold increase at PWP in sandy loam soil. In contrast, our results revealed a less pronounced effect of ASi on the FC, PWP, and available water capacity (AWC) of silty clay soil. One may attribute this effect to the distinctive properties of ASi, allowing it to absorb a substantial volume of water onto its surface. ASi particles exhibit a significant specific surface area (270–330 m^2^ g^−1^), stemming not only from their relatively small diameter (within the nanometer range) but also from their noteworthy internal porosity. The term “internal porosity” denotes the presence of pores within the ASi particles themselves. However, it’s worth noting that if this phenomenon were the sole governing factor, we would have expected a similar effect of ASi on the water-holding capacity of both soils. The most credible explanation for the observed phenomena in sandy loam soil lies in the influence of ASi on soil pore size distribution. The particle size of ASi, falling within the nanometer range (ca. 7 nm), is considerably smaller than the typical soil particles found in sandy soil. This disparity in size has the potential to alter the pore size distribution in coarse-textured soil, specifically shifting it towards smaller pore sizes with a broader size distribution^29,30^. In other words, adding ASi to coarse-textured soil shifts the pore size distribution toward fine-textured soil^31^. Consequently, this shift ensures a gradual drainage of pores in response to a drop in matric potential. This shift in the pore size distribution of ASi-treated sandy loam is also evident in a lower observed ${K}_{h}$ at FC of sandy loam (Table 1). The ASi-treated sandy loam had 25-fold lower ${K}_{h}$ at FC than the control soil. However, if we define plant available water content (AWC) as the difference between FC and PWP, the application of ASi had no increased effect on AWC since it increased both FC and AWC at the same rate (Table 1) as water contents at both FC and PWP were increased.

In this study, we investigated the impact of ASi on water flow within the soil by examining the ${K}_{h}$ (Fig. 1). Let’s concentrate on examining the effect of ASi on the ${K}_{h}$ of both soils within the dry soil range. This focus is essential because the Hyprop technique, employed for ${K}_{h}$ measurements, demonstrated limited sensitivity in the wet soil range. Moreover, noteworthy soil swellings were observed during the measurements of saturated hydraulic conductivities in ASi-treated soils, particularly when the soil samples were initially sturated. By narrowing our focus to the dry soil range, we aim to obtain a clearer understanding of ASi’s influence on hydraulic conductivity without the complications observed during saturated conditions and target our discussion to the range that soil hydraulic conductivity becomes crucial for root water uptake^2^. Typically, ${K}_{h}$ decreases as soils dry. However, our findings demonstrate that the presence of 2% ASi in sandy loam soil (representative of coarse-textured soil) prevented a significant drop in ${K}_{h}$ as the soil dried, maintaining a 5.13 and 4.3-fold higher ${K}_{h}$ compared to the control soil at soil matric potential of − 4000 cm (h4000) and PWP, respectively. A similar effect was observed in silty clay, but the magnitude of this effect was diminished (1.8-fold increase) at a h4000 and more pronounced (7.67-fold increase) at PWP. The selected soil matric potential of − 4000 cm is utilized herein as a representative indicator of soil water potential at which wilting symptoms were manifested in our plants, deduced from the recorded soil water potential values on the final day of measurement (Fig. 4).

To explain this effect, let’s examine the factors that affect ${K}_{h}$. ${K}_{h}$ is a function of soil water content, size distribution of water-filled pores, and the connectivity of the liquid phase. A higher soil water content signifies not only a larger cross-sectional area available for water flow but also offers a shorter flow path within larger pores where less friction is imposed on the water as it moves within the soil (as the soil dries, remaining water is constrained to smaller pores). Both of these phenomena result in a higher ${K}_{h}$. We attribute the effect of ASi on ${K}_{h}$ of sandy loam soil to its effect on pore size distribution and shifting them to a smaller range, which in turn increased water holding capacity of soil at any given water potentials. While this shift may reduce ${K}_{h}$ of sandy loam soil at near-saturated conditions due to higher expected friction on the water within pores, our results show a higher ${K}_{h}$ in the presence of ASi in the dry range, indicating that the effect of ASi on increasing water holding capacity and increasing the connectivity of the liquid phase was dominant. Typically, liquid connectivity, which is inversely proportional to soil water content (with an exponent bigger than one), is interrupted as the soil dries. This interruption is more pronounced in coarse-textured soils (hosting large pores) as soil matric potential drops and a significant fraction of larger pores drain. Shifting the pore size distribution towards smaller sizes delays such interruption. Shift in pore size distribution results in a gradual drainage of pores as matric potential drops. The additions of 2% ASi in silty clay soil reduced ${K}_{h}$ in matric potentials ranging from − 100 to − 5000 cm. This can be explained by further reduction of pore size distribution towards smaller sizes, resulting in higher friction on water from soil pore walls.

Driven from observations, our modeling results suggest that ASi treatment delays transpiration reduction in sandy loam soil to a lower range of soil matric potential (Fig. 5b) and allows plants to explore more negative bulk soil and leaf water potential (Fig. 5a,b) than the control. In contrast, ASi treatment had no significant effect on soil–plant–water relations of silty clay soil. As tomato plants were chosen for the current experiment, which are Si excluder plants, we can relate the effects of ASi on soil–plant–water relations in the sandy loam soil to processes occurring in the soil. Based on our result, we conclude that the plants grown in sandy loam soil treated with 2% ASi had easier access to water than those grown in control, enabling them to experience a more negative soil and leaf water potential. In contrast, those plants grown in the control treatment had to reduce their transpiration due to limited water flow within the soil. We attributed this positive effect in sandy loam to the effect of ASi on ${K}_{h}$. At first glance, the presence of ASi in sandy loam soil seems to reduce soil matric potential at any given water content compared to the control soil, which can make water less available to plants as water is held tightly within the soil pores. This effect is also reflected in the predawn leaf water potential measurements, as plants grown in sandy loam soil treated with 2% ASi exhibited much lower leaf water potential than those grown in the control soil (here we assumed that at predawn, both leaf water potential and soil water potential are similar due to low plant transpiration). However, contrary to this perspective, the facilitation of water flow within the soil and particularly at the root-soil interface, due to the effect of 2% ASi on ${K}_{h}$, allowing the plants grown in sandy loam soil to delay their transpiration reduction to a lower range of soil matric potential and explore more negative leaf water potential, as evidenced by Fig. 5a,b. In sandy soil, the application of 2% ASi induced a consistently higher ${K}_{h}$ relative to the control, beginning at a soil matric potential of approximately − 300 cm. This treatment resulted in a significant increase in ${K}_{h}$, by a factor of 5.13, at a soil matric potential of − 4000 cm. This specific matric potential is identified as a critical indicator of soil water potential correlating with the onset of wilting symptoms in our plants, as inferred from the soil water potential measurements recorded on the ultimate day of observation. Conversely, in silty clay soil, the 2% ASi treatment enhanced ${K}_{h}$ compared to the control, starting from a soil matric potential of approximately − 2000 cm. This enhancement maintained the ${K}_{h}$ of silty clay at a level 1.8 times higher than that of the control at − 4000 cm. Nevertheless, this increment appeared insufficient to significantly affect plant transpiration. However, under conditions where plants could exploit more negative water potentials (e.g., up to PWP), application of 2% ASi might improve soil–plant–water relation in silty clay soil as well since the ${K}_{h}$ of silty clay exhibited a 7.67-fold increase at PWP.

Finally, it is important to note that the conclusions drawn regarding the impact of ASi on soil–plant water relations, as derived from our modeling exercise, assume that the hydraulic properties of soils in our planted pots were identical to those measured in unplanted soils mixed with ASi. However, existing literature has consistently demonstrated that the presence of roots and their activities can significantly alter hydraulic properties^32^, a factor that was neglected in our study. Moreover, it is essential to highlight that while our soils showed varying initial ASi content—the sandy loam containing 0.3 mg and the silty clay 0.72 mg of ASi per gram of dry soil—this difference proves inconsequential when factoring in the application rate of 2% ASi. This consideration ensures that the nominal difference in initial ASi content between the two soil types exerts minimal influence on the comprehensive evaluation of ASi effects within the scope of this study.

## Conclusion

In conclusion, our comprehensive investigation into the influence of ASi on soil–plant–water interactions across different soil textures reveals significant outcomes. The application of 2% ASi has a pronounced impact on sandy loam soil, enhancing water retention at FC and PWP by factors of 1.33 and 3.5, respectively, while exhibiting a negligible effect on silty clay soil. Our study further explores the implications for plant access to water, indicating that tomato plants in ASi-treated sandy loam soil sustain higher transpiration rates under drying conditions compared to the control. Modeling results corroborate these observations, suggesting that ASi treatment delays transpiration reduction in sandy loam soil, allowing plants to access water at more negative matric potentials. In contrast, ASi demonstrates a limited impact on soil–plant–water relations in silty clay soil. While ASi may initially reduce soil matric potential in sandy loam soil, facilitating water retention, its overall effect on water flow at the root–soil interface enables plants to explore more negative leaf water potential. This dual impact of ASi on water retention and enhanced hydraulic conductivity contributes to improved water availability for plants in coarse-textured soils. In summary, our findings underscore the potential of ASi to optimize soil–plant–water dynamics in specific soil types, offering valuable insights for sustainable agricultural practices.

## Materials and methods

### Soil preparations

The impact of ASi on soil–plant water relationships was assessed through a pot experiment with a randomized design. The experiment involved four treatments: two soils of contrasting textures (sandy loam and silty clay) and two ASi contents (0 and 2 g ASi per 100 g of oven-dried soil). The decision to use a 2% ASi application rate was informed by the findings of Zarebanadkouki et al.^19^, who investigated the impact of different silicate forms at varying concentrations (0, 1, and 5%) on the retention curve of a sandy soil. Initially, we intended to opt for a 1% ASi content for economic reasons. However, preliminary tests indicated only marginal effects on the retention curve and hydraulic conductivity of both soils at this application rate. In contrast, an application rate of 2% exhibited significant and noteworthy effects on the hydraulic properties of soils. The soils were collected from A horizon of two soils in Bayreuth, Germany. The sandy loam had 19% clay, 8% silt, 73% sand (determined based on a sedimentation method implemented by a commercial device called Pario^33^, and 0.8% organic matter (OM), and silty clay soil had 41% clay, 44% silt, 15% sand, and 1.6% OM. The sandy loam had 0.3mg ASi per g of dry soil and silty clay 0.72 mg ASi per g dry soil, determined by the Tiron-method^22^. The ASi used in the experiment was hydrophilic ASi in the form of oven-dried Aerosil 300 (from Evonik Industries AG, Essen, Germany). This ASi had a specific surface area of 270–330 m^2^ g^−1^, mean particle diameter of 7 nm, and particle density of ca. 0.5 g cm^−3^. The soils were first air-dried, then sieved through a 2 mm mesh size, and finally mixed uniformly with the Si at a ratio of 0 or 2 g ASi per 100 g of oven-dried soil.

The effect of ASi on soil water retention and flow was evaluated by measuring soil water retention and hydraulic conductivity curves. The soil retention curve and hydraulic conductivity curve of the soil mixtures were determined using the evaporative method implemented by the Hyprop device (Meter Group, Munich, Germany). This device comprises two tensiometers and a balance connected to a computer to monitor soil water potential at two depths and average soil water content during soil drying. The soil samples were packed into Hyprop sample holders of 50 cm^2^ area and 5 cm height (three replications) at the same bulk density as the experimental pots used for plant growth (values are given in the Plant preparations). The soil was saturated for 48 h and then allowed to dry via evaporation from the top while monitoring soil matric potential, average soil water content, and evaporative flux over time. Hyprop cannot estimate soil hydraulic conductivity near saturation, so the saturated hydraulic conductivity was quantified using the falling head method using the KSAT device (METER Group Inc.). The soil retention and hydraulic conductivity curves were finally modeled based on the data from the Hyprop and KSAT experiments using the Peters–Durner–Iden (PDI) model^34^.

### Plant preparations

In this study, tomato plants (*Solanum lycopersicum* L.) were used as model plants which (i) are known to exclude silicon and have a low accumulation of silicon in their aboveground biomass^27^, (ii) develop a sizeable aboveground biomass with high transpirational demand, allowing us to remove a few leaves during the soil drying cycle to assess plant water status without affecting their transpiration rate. The M82 tomato variety was used in this experiment. The seeds of this variety were obtained from Rootility® (Israel).

The seeds were initially subjected to a 30-s hydrogen peroxide treatment and then allowed to germinate in the dark for 5 days on a moist filter paper. The seedlings were then grown in 30 cm tall and 9 cm diameter polyvinyl chloride (PVC) cylinders. The PVC pots were filled with air-dried soils by passing soil through a sieve (2 mm mesh) to ensure uniformity and avoid layering within the PVC pots. During the soil filling process, a tensiometer (TEROS 21, METER Group, Inc. USA) was placed in the center of each PVC pot (at a depth of 15 cm). This resulted in an average soil bulk density of 1.40 and 1.46 g cm^−3^ for the sandy loam and silty clay soils without ASi (control), respectively, and of 1.23 and 1.13 g cm^−3^ for the sandy loam and silty clay soils mixed with 2% ASi, respectively. This reduction is partly expected due to the lower density of ASi particles (0.5 g cm^−3^) compared to typical soil particle density (2.65 g cm^−3^). The PVC pots had drain holes at the bottom to remove excess irrigation water. Five holes (with a diameter of 5 mm) were placed at depths of 5, 10, 15, 20, and 25 cm to measure soil water content using a time-domain reflectometer (TDR; Easy Test, Poland, with 0.1% reading resolution). The TDR was calibrated for the studied soils.

For each treatment (texture and ASi contents), six seedlings (replications) were planted at a depth of 1.5 cm in the center of each PVC pot. During the growth period, the plants were maintained inside a climate chamber with a night/day temperature of 18/25 °C, relative humidity of 67/63%, and a daylight intensity of 1100 μmol m^−2^ s^−1^. At 10 days old (following plantation), the surface of each pot was covered with a 1 cm layer of coarse sand to decrease evaporation. Plants were regularly irrigated with water mixed with a nutrient solution to maintain optimal soil water content within 0.35–0.25 cm^3^ cm^−3^.

When the plants were ca. 60 days old, the pots were irrigated by a capillary rise from the bottom (the water table was set at 10 cm) for 24 h to ensure uniform and consistent water content across different replicates. This procedure resulted in an average water content of 0.31 ± 0.02 and 0.32 ± 0.01 cm^3^ cm^−3^ in sandy loam treated without and with ASi, respectively, and 0.36 ± 0.02 and 0.39 ± 0.01 cm^3^ cm^−3^ in silty clay treated without and with ASi, respectively. Afterward, the pots were removed from the water and allowed to drain excess water by gravity for 6 h. Then, irrigation ceased, and the plants could dry the soil. During this soil drying cycle, the PVC pots were placed on a digital balance connected to a computer to monitor their weights over time (at 10-min intervals) to estimate their transpiration rate as a function of time and soil water contents.

### Transpiration measurements

The plant transpiration rates were calculated by determining the change in plant weight between two consecutive measurements over a 10-min interval, assuming that the difference in plant weight and water loss from the soil surface was negligible. Note that the soil surface was covered with a 1 cm layer of coarse sand to decrease soil evaporation. Since the plants had varying leaf areas, the transpiration rate was normalized per the maximum transpiration rate recorded on the first day of the drying cycle.

### Soil water content, soil water potential, and leaf xylem water potential measurements

During the soil drying cycle, soil water content was measured at five different depths (intervals of 5 cm) using a TDR, and soil water potential was measured at a depth of 15 cm in the middle of the soil using a TEROS-21 sensor that was placed in the middle of experimental pots. The xylem water potential of the transpiring leaves was measured using a Scholander pressure chamber. A section of the leaf (approximately 2 cm long) was cut and placed in a dark plastic bag for 45 min^35^. The leaf was then sealed in the pressure chamber, and the air pressure gradually increased until a drop of water was visible at the cut edge of the leaf. The pressure required to maintain the drop of water at the cut end of the leaves is numerically equivalent to the tension in the xylem^9^ and is referred to as the leaf water potential. This measurement was taken at 5:00 a.m. (when transpiration is low), 10:00 am, and 2:00 pm (at the peak of transpiration). The leaf water potential measured at 5:00 am and 2:00 pm will be referred to as the predawn leaf water potential and midday leaf water potential, respectively. Note that we consistently observed some transpiration during nighttime, and the term predawn was selected here to refer to water potential when transpiration reached its lowest value. The leaf water potential was measured at days 1, 3, 4, 5, 6, days and 100 after the soil drying cycle started in sandy loam soil and at days 1, 2, 3, 4, 5, and 6 in silty clay soil. Note that both soils had relatively similar initial water content at the beginning of the soil drying cycle, but plants reached their wilting point earlier and in wetter soil in the case of silty clay than sandy loam.

### Soil–plant hydraulic model

To test the hypothesis that ASi facilitates root water uptake during soil drying, we simulated water flow across the soil–plant–atmosphere continuum (SPAC). Inspired by the work of Sperry and Love^7^ and Carminati and Javaux^2^, a soil–plant hydraulic model was used to simulate the relation between soil water potential, leaf xylem water potential and the transpiration rates during soil drying. This simplified model describes the water flow across the soil–plant–atmosphere continuum (SPAC), representing the SPAC as a series of hydraulic resistances between the plant leaves and bulk soil (acting as capacitance). The flow of water is assumed to be steady state, namely flow rates (cm^3^ s^−1^) across each component of SPAC between the bulk soil and the leaves (i.e., soil, root, xylem, leaf) are assumed to be equal. The model is described in detail in Carminati and Javaux^2^. In brief, water flow across each flow domain is driven by the gradient in water potential and regulated by the respective domains’ hydraulic conductance. Under steady rate assumption, the radial flux of water within soil can be described by

1 $$q\left(r\right)=-{k}_{s}\left({\psi }_{s}\right)\frac{\partial {\psi }_{s}}{\partial r}$$

where $q\left(r\right)$ is water flux within soil [cm s^−1^] at any given radial distance of $r$ from root surface, ${\psi }_{s}$ is the soil matric potential expressed in meter heads [1 cm ≈ 1 hPa], *r* is the radial coordinate [cm] and ${k}_{s}$ is the soil hydraulic conductivity [cm s^−1^]. Assuming a uniform root water uptake rate along the entire root system, the radial flux of water at any given radial distance of $r$ from the root surface within the soil can be calculated as

2 $$q\left(r\right)=-\frac{E}{2\pi rL}$$

where $E$ is plant transpiration rate [cm^3^ s^−1^], and $L$ is active root length in water uptake. Note here replacing $r={r}_{0}$ gives the root water uptake rate at the root surface. For simplicity and aiming to derive an analytical solution for Eq. (1), the ${k}_{s}\left({\psi }_{s}\right)$ is defined as

3 $${k}_{s}\left({\psi }_{s}\right)={k}_{sat}{\left(\frac{{\psi }_{s}}{{\psi }_{s,0}}\right)}^{-{\tau }_{s}}$$

where ${k}_{sat}$ is saturated hydraulic conductivity of soil [cm s^−1^], ${\psi }_{s,0}$, ${\tau }_{s}$, are fitting parameters. Let us substitute Eqs. (2) and (3) in Eq. (1) and define matric flux potential [$\varnothing \left({\psi }_{s}\right)$, cm^2^ s^−1^] as

4 $$\varnothing \left({\psi }_{s}\right)=\underset{{\psi }_{, lo}}{\overset{{\psi }_{, up}}{\int }}{k}_{s}\left({\psi }_{s}\right)d{\psi }_{s}$$

where ${\psi }_{, lo}$ and ${\psi }_{, up}$ are lower and upper range of integration. Such consideration allows the derivation of a linearized analytical solution for Eq. (1) based on Kirchhoff transformation approach (see Carminati and Javaux^2^ for more details). Similarly, flow of water across the root system can be described by

5 $$E={-K}_{root}\left({\psi }_{x,r}-{\psi }_{sr}\right)$$

where $${K}_{root}$$ is the root hydraulic conductance [cm^3^ MPa^−1^ s^−1^], $${\psi 
}_{x,r}$$ is the root xylem water potential [MPa], and $${\psi }_{sr}$$ is the soil water potential at the root surface [MPa]. Finally, the flow equation within the xylem from roots to the leaves is represented as

6 $$E={-K}_{x}\left({\psi }_{x,l}\right)\left({\psi }_{x,l}-{\psi }_{x,r}\right)$$

where ${K}_{x}$ is the xylem hydraulic conductance [cm^3^ MPa^−1^ s^−1^], and ${\psi }_{x,l}$ is the leaf xylem water potential within plants [Mpa]. Let us assume xylem cavitation in the range of observed water potentials is negligible and then replace ${K}_{x}\left({\psi }_{x,l}\right)$ by ${K}_{root}.$

### Model parameterization and inverse modeling

These equations were analytically solved using Python to determine the soil water potential profile as a function of distance from the soil surface, root xylem water potential, and leaf xylem water potential under varying soil drying conditions and plant transpiration rates. To begin with, a 1D radially symmetric flow domain in the soil was constructed, with the radial distance ${r}_{b}$ corresponding to the maximum distance between roots, given by

7 $${r}_{b}=\sqrt{\frac{{V}_{s}}{2\pi L}}$$

where ${V}_{s}$ is the total volume of soil [cm^3^], and $L$ is the total length of active roots involved in water uptake [cm]. Next, the fitting parameters in Eq. (3) (${\psi }_{s,0}$, ${\tau }_{s}$) were determined for each soil by fitting this equation to the measured data of the soil hydraulic conductivity curve (measured by Hyprop method). The ${K}_{root}$ in Eq. (5) was determined by fitting a linear equation (with zero intercept) through the measured data of $E$ and ${\psi }_{x,l}$ on the first day of the experiment, when the soil was still wet. This decision was made based on the assumption that t the hydraulic conductance of the roots primarily governs the plant transpiration rate in wet conditions. Finally, Eqs. (1), (5), and (6) were simultaneously solved by imposing soil matric potential and plant transpiration rate as boundary conditions and then inversely adjusting the value of $L$ to best reproduce the measured relation between $E$ and ${\psi }_{x,l}$ for any given ${\psi }_{s}$. To achieve this goal, an objective function was defined to quantify the goodness of fit as the sum squared difference between the observed and model-simulated $E$ and ${\psi }_{x,l}$. To minimize the objective function, the Nelder algorithm, implemented in the LMfit package for Python, was employed. The Nelder–Mead algorithm iteratively adjusts the value of L to minimize the predefined objective function.

Carminati and Javaux^2^ defined the onset of plant stomata closure based on detecting a trajectory that separates the relation between $E$ and ${\psi }_{x,l}$ into linear and nonlinear parts. They assumed that plants should close their stomata near this threshold as beyond this threshold the ${\psi }_{x,l}$ is decreasing significantly for a small increase of $E$. In practice, this threshold at each ${\psi }_{s}$ is determined as simulated ${\psi }_{x,l}$ where $\left|\frac{\partial E}{{\partial \psi }_{x,l}}\right|\le 0.75{\left|\frac{\partial E}{{\partial \psi }_{x,l}}\right|}_{max}$.

### Statistical analysis

To assess the statistical significance of the treatments (the ASi content), in influencing the hydraulic properties of soils (Table 1), a one-way ANOVA test was performed for each dependent variable using the Pingouin package in Python. Following this initial analysis, a t-test was applied to evaluate significance levels at both p = 0.05 and 0.01. The measured leaf water potentials and normalized transpiration rates across different replications of each treatment were used first to construct a continuous line that describes the relationship between plant-normalized transpiration rates and leaf water potentials. Note that combination of all replications together was necessary to ensure a reliable fit across varying rang of soil water contents. This relation was achieved by solving water flow equations within soil–plant systems. Subsequently, to enhance the robustness of the analysis, 95% confidence intervals of the fits were calculated. The statistical significance of each treatment, concerning both plant-normalized transpiration and leaf water potentials, was tested based on the optimal fit values and their corresponding 95% confidence intervals. If the confidence intervals do not overlap, we assumed that there is a significant difference between two tested variables.

### Plant guideline statement

The experiment used seeds of the M82 tomato variety sourced from Rootility® (Israel). This variety is not listed among Germany’s national and provincial key protected wild plants, nor is it identified as a threatened species on the IUCN Red List. Consequently, no specific permissions or licenses were required for plant sampling for research purposes. We affirm our adherence to the IUCN Policy Statement on Research Involving Species at Risk of Extinction and the Convention on the Trade in Endangered Species of Wild Fauna and Flora.

## Data Availability

The datasets used and/or analysed during the current study are available from the corresponding author on request.
